# Application of Crowd Simulations in the Evaluation of Tracking Algorithms

**DOI:** 10.3390/s20174960

**Published:** 2020-09-02

**Authors:** Michał Staniszewski, Paweł Foszner, Karol Kostorz, Agnieszka Michalczuk, Kamil Wereszczyński, Michał Cogiel, Dominik Golba, Konrad Wojciechowski, Andrzej Polański

**Affiliations:** 1 Department of Computer Graphics, Vision and Digital Systems, Faculty of Automatic Control, Electronics and Computer Science, Silesian University of Technology, Akademicka 2A, 44-100 Gliwice, Poland; pawel.foszner@polsl.pl (P.F.); kostorz.karol@gmail.com (K.K.); agnieszka.michalczuk@polsl.pl (A.M.); kamil.wereszczynski@polsl.pl (K.W.); michal.cogiel@polsl.pl (M.C.); dominik.golba@polsl.pl (D.G.); andrzej.polanski@polsl.pl (A.P.); 2 Polish-Japanese Academy of Information Technology, Koszykowa 86, 02-008 Warszawa, Poland; konrad.wojciechowski@polsl.pl

**Keywords:** tracking multiple objects, visual object tracking, urban data collection, video acquisition, image processing, crowd simulations, multiple people tracking, benchmark

## Abstract

Tracking and action-recognition algorithms are currently widely used in video surveillance, monitoring urban activities and in many other areas. Their development highly relies on benchmarking scenarios, which enable reliable evaluations/improvements of their efficiencies. Presently, benchmarking methods for tracking and action-recognition algorithms rely on manual annotation of video databases, prone to human errors, limited in size and time-consuming. Here, using gained experiences, an alternative benchmarking solution is presented, which employs methods and tools obtained from the computer-game domain to create simulated video data with automatic annotations. Presented approach highly outperforms existing solutions in the size of the data and variety of annotations possible to create. With proposed system, a potential user can generate a sequence of random images involving different times of day, weather conditions, and scenes for use in tracking evaluation. In the design of the proposed tool, the concept of crowd simulation is used and developed. The system is validated by comparisons to existing methods.

## 1. Introduction

Person tracking and action-recognition algorithms for video streaming data recently focus a lot of interest, inspired and motivated by variety of possible applications. There are numerous approaches and achievements, often multistage and complicated, leading to different types of information extracted from data. Their further improvement and evolution require elaborating reliable evaluation and benchmarking scenarios. Existing benchmarking methods involve testing the reliability of tracking and action-recognition algorithms by employing public datasets manually annotated by their authors. PETS [1] is the first, well-known dataset created primarily for surveillance applications. The original release consisted of three subsets of benchmark data, the first designed for pedestrian count and density analysis, the second for pedestrian tracking, and the last for analysis of traffic flow and event recognition. With respect to evaluation of tracking methods, the most popular dataset is included in MOTChallenge [2,3,4,5], which includes available datasets with ground truth, validation MATLAB scripts and the possibility of uploading results so as to rank methods with respect to quality and accuracy.

However, manual preparation of annotations of benchmarking data leads to substantial limitations and shortages. The first limitation concerns data volume and resolution, which clearly must be downsized due to constraints imposed by “throughput” of human operators. The second limitation is insufficient replicability and possible biases introduced by disagreements between annotations of different human experts (operators). These limitations and shortages lead to biases and errors in evaluating algorithms for tracking and action-recognition [2]. Without sufficient variety and replicability of benchmarking data evaluations of tracking and action-recognition algorithms are likely to overestimate their efficiencies due to the phenomenon of multiple testing with overfitting of design parameters. Limitations of existing benchmarking approaches lead many authors to create their own testing data records, a challenging and time-consuming task [6]. In order to prepare such a dataset, authors have actors perform specially designed scenarios and then they again manually annotate where and what actions were performed. However, such in home prepared benchmarks typically lead to severe problems in their lack of standardization and/or irreproducibility [4,5]. Limitations of manually prepared data were also observed by the authors of this paper, during work on practical implementation of the surveillance project [7].

The above described problems justify the need for conducting serious research towards elaborating machine—generated benchmarking systems of large variability, resolution and volume. Methodologies for developing benchmarking methodologies for person tracking and action-recognition algorithms must address challenges related to the need for elaborating applications for many different tracking [8] and action-recognition [9] environments, including the analysis of surveillance video and urban data understanding. The desired task is to track multiple objects employing simple and compound action recognition and many other aspects of computer vision. The quality of these analyses can be influenced by variations in weather conditions, lighting levels, occlusions of people, and changes in camera position. Algorithms, which have proved their usefulness in such analyses over the last decade, now must deal with relatively larger datasets than previously due to optimization and the rapid growth of computational power [5].

Realistic behavior and motion can be generated by employing the general concept of a crowd simulator, a piece of software that allows a user to determine the movement and behavior of a user-defined number of crowd members, who are often referred to as actors, or agents, in specific, variable circumstances [10,11,12,13,14,15,16]. Crowd simulators can be divided into two main categories: those that represent autonomic crowd behaviors in the most realistic and faithful way and those that aim to produce persons/groups visualizations of the highest possible graphical fidelity for visual human reception. The primary use of simulators belonging to the first category is to aid in the design of effective evacuation routes from buildings or during mass events. Realistic simulations of crowd behavior allow testing of safety solutions cost effectively in a contained environment, further improving their quality. Examples of such simulators are the MASSIS framework [17] and the PedSim, available as a C++ library [18]. Agent positions generated by PedSim can be used in a user’s graphical engine of choice to produce visualizations. While a crowd’s behavior can typically be modeled with high accuracy in this type of simulator, the visual side of things is treated in a purely utilitarian way, often too minimalistic or overly simplified (or simply lacking) for the needs of computer vision. The second category of simulator is used primarily to create visual effects in movies and animations. Simulators of this type allow a scene to be populated with realistically looking and behaving virtual actors. The most popular professionally used simulators of this type are the Golaem Crowd [19], Miarmy (Autodesk Maya plugin), and the MASSIVE environment [20]. Such software can produce high-quality visualizations, but the crowd agents often lack autonomy and customizability.

W. van Toll, M. et al. [21] defines a five-level hierarchy for path planning in crowd simulation systems. This hierarchy allows convenient division of all necessary tasks required to create realistic motion in crowd simulation systems. As of yet, many video game engines, including Unity, Unreal Engine, or CryEngine, provide useful, convenient, and sufficient tools for path planning and overall control of agents during route following. Unity game engine enables its NavMesh Agent [22] to address this issue. Kristinsson [23] describes a system based on Unity 3D that along with a properly set animation controller component and adequate animation clips, can employ motion–capture technology to record video clips and high-resolution 3D models, thereby creating a realistic crow simulation system. In one way to approach this problem, Forbus et. al. [24] describe a video game entitled “The Sims” created by Maxis Studios which gives a player the ability to create and control a virtual family over many generations. Agents, or sims, can perform various actions to achieve objects worldwide or with one another. Actions performed by agents, or indirectly controlled Sims, occur when a user creates a temporal-interaction object with a proper animation clip or animation offset in order to impart the feel of a real-world situation. To achieve such an effect, every action needs to be manually defined by the user. The Sims comes with a virtual machine, The Edith, designed specifically for this purpose. Musee et al. [25] propose a different approach in which a crowd simulator simulates the motion of synthetic pedestrians drawn from samples gathered from real-world video sequences using object tracking techniques. The simulator uses the trajectories of pedestrians gathered from video sequences to simulate pedestrians moving within a simulated environment. In [26], the authors report creation of an interactive data-driven crowd simulator that combines the high realism of data-driven methods with interactivity of synthetic techniques.

As previously mentioned, crowd simulators have already numerous applications including not only gaming software but also city and environments planning, organizing commercial spaces, designing and verification of evacuation paths for buildings or terrains etc. All these applications inspire fast advances in crowd simulators. In given work a new application of crowd simulators is presented. The CrowdSim (crowd simulation system) was designed and employed as a validation tool for tracking video recordings. To the best of the authors knowledge, CrowdSim is the first system of this type that allows generation of random test data with high variability and controlled complexity.

A graphical system is designed that creates simulated records together with matching annotations. With this system, a potential user can generate a sequence of random images incorporating differing times of day, weather conditions, and scenes for the purpose of tracking evaluation. Published algorithms can thus be tested with respect to the influence of crowd density or the impacts of different light conditions related to weather, both of which are difficult to capture when real-world videos are employed. The proposed solution was tested and validated on different algorithms for tracking multiple objects and these results were compared with those obtained from MOTChallenge. The presented work has the following features:

- random generation of images that can be used to evaluate different algorithms for tracking multiple objects by means of random starting position of pedestrians, their unpredictable interactions during simulation process and choice of different models
- application of the Unity game engine for crowd simulation
- automatic annotation of object detections and names of actions according to the MOT format
- application of prepared models, generated scenes, and changes in time of day and weather conditions during a simulation

## 2. Materials and Methods

### 2.1. Proposed Solution—CrowdSim

**Figure 1:**
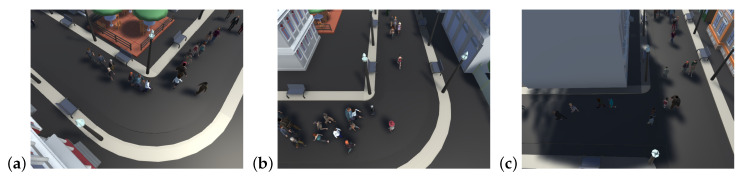
Example of crowd simulations of CrowdSim given in three different views—(**a**–**c**) three different cameras.

**Figure A1:**
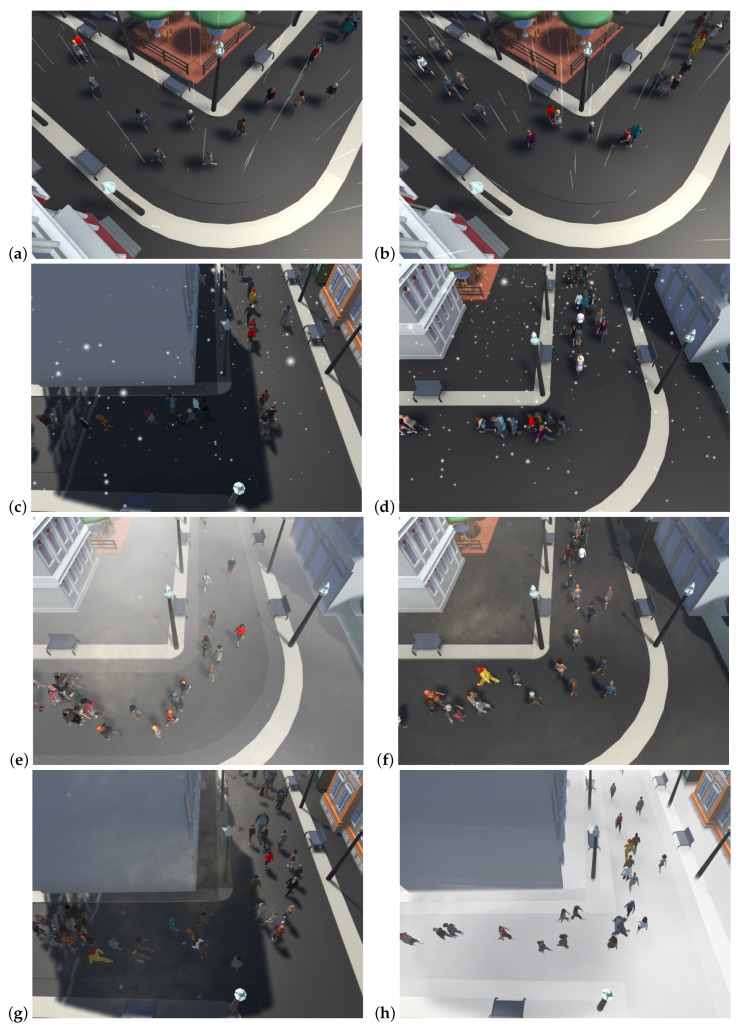
The influence of different weather conditions with various intensity defined by the user: rain (**a**,**b**), snow (**c**,**d**) and fog/steam (**e**–**h**).

The purpose of proposed crowd simulator (CrowdSim) is testing multi-object tracking algorithms. Moreover, it was intended to be a worthy replacement for datasets of existing video footage and to introduce a new level of testing for tracking algorithms by generating simulations with a variety of factors that would not be possible using real-world camera footage. The CrowdSim is based on the very popular Unity 3D engine that offers excellent optimization and ease of development. Figure 1 (and additionally Figure A1), drawn from a creation of proposed simulator, shows three scenes representing views from three cameras focused on a closed city square. Presented solution is equipped with three different views (static cameras) but the number of possible scenarios is very high—future user can change (1) number of pedestrians in scene, (2) number of FPS (Frame Per Second) and resolution, (3) intensity of different weather conditions (rain, fog and snow). Simulations are compatible with MOTChallenge format and generated along with ground truth (exact results of crowd identification), detections which are the necessary information of detected objects, but not yet marked as individual objects and finally generated images in png format.

The presented approach employs not only the possible combinations of video environments available to a user but also allows that user to test algorithms capable of tracking objects via footage from multiple cameras simultaneously. The user can track an object if it leaves one camera frame but appears in the field of view of another. The main challenges in implementation of CrowdSim rely on generation of 3D models along with animations and creation of assumptions concerning agent’s movement.

In addition, CrowdSim offers a variety of models, 50 different ones in total (25 male and 25 female characters) created with the Adobe Fuse CC tool. This amount of variety makes the simulation more reliable when it is testing algorithms based on agents’ appearances. The Mixamo tool emulates walk animation of human figures, resulting in very natural looking agent movement. To achieve natural pedestrian distribution and walking paths, the authors introduced random initial positions of pedestrians across a scene. In addition, Crowd Simulator with the Unity engine was employed to create a net of points of interest for agents to ensure they move around the simulated city square in a natural and predictable manner. Due to the many interactions between agents during the simulation (e.g., occlusions, path intersections in crowded places), which slow down or stop the agents, their movement is not uniform and makes the tracking process more difficult.

**Figure 2:**
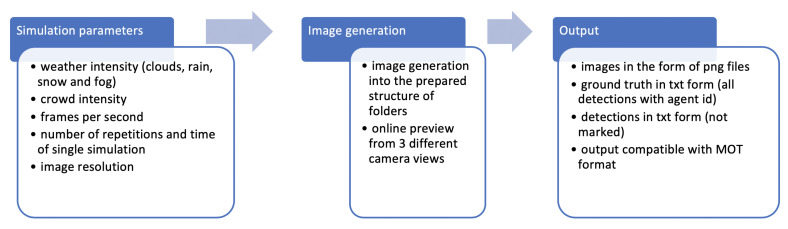
Possible workflow of CrowdSim with respect to each step: setting simulation parameters, automatic image generation and output.

The primary features of Unity, including its usage of an A-star [27] algorithm for global path finding or of reciprocal velocity obstacles to represent collision predictions or collision avoidance, are sufficient to create realistic movement among agents. Other useful functions include constant access to each agent’s current and desired velocity and the ability to prioritize an agent’s movement to define its deviation from a planned route. What makes this approach special is a mechanism that saves information about an object’s position in every frame in a format already popularized by MOTChallenge, a characteristic that makes using this simulator as a test environment for tracking algorithms incredibly easy. Still another useful addition is the potential to simulate various weather conditions and their intensity. Possible workflow of CrowdSim is presented in Figure 2.

### 2.2. Evaluation Scheme

In verification of CrowdSim the MATLAB computational environment has been used as the tool for organizing and ordering experiments as well as for statistics. The material for analysis came from 6 different tracking algorithms that are available along with open-source code. Analysis of the system evaluation was performed in MATLAB (The MathWorks, Natick, MA, USA) on two different workstations, a 2.6-GHz Inter Core i7 with 16 GB RAM and a 3.5-GHz 6-Core Intel Xeon E5 with 64 GB RAM but without GPU acceleration, using standard MATLAB libraries and up to six workers. Execution of tracking algorithms was evaluated separately based on the source code available and was performed on a computer equipped with 3.6-GHz Intel Core i7, NVidia GeForce GTX 1070, and 32-GB RAM. The sample images along with source code are provided online at (https://crowdsim.aei.polsl.pl/). In order to automate the work related to testing multi-object algorithms, a test environment was created, and the main tasks of a testing environment included the following:

- adaptation of the input data to suit the input format of each algorithm
- conversion of algorithm outputs to the form standardized by MOTChallenge
- automation of triggering algorithms in the form of a pipeline
- harvest of standardized outputs and performance ratings with use of the MOTChallenge workspace
- workspace clean-up and preparation for another run

**Figure 3:**
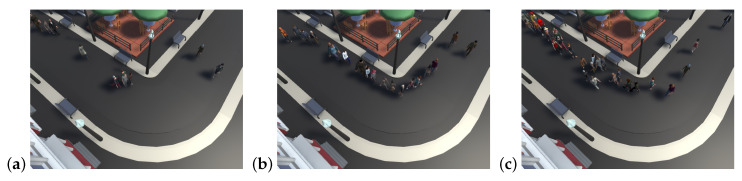
Example of crowd simulations for 10 (**a**), 50 (**b**) and 100 (**c**) pedestrians walking on the simulation in one camera view used in analysis of influence of crowd density on tracking algorithms.

The dataset was split into two categories: one to measure crowd density, variety, and performance and the other to evaluate the impact of weather conditions. In both cases, data were acquired from three camera angles. Performance according to the crowd density is given in Table 1 and Figure 3.

**Figure 4:**
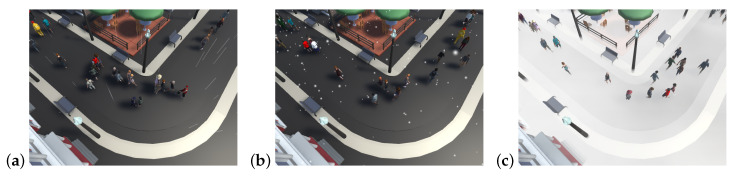
Crowd simulations of one camera view to analyze weather influence on tracking algorithms for three different weather conditions: rain (**a**), snow (**b**), and fog (**c**).

The frequent occlusion effect caused by weather conditions increased the randomness of results in the weather-related test. To make full use of the crowd simulator chosen for this task, data were generated with every weather condition possible according to parameters given in Table 2 and Figure 4. Both rain and snow were applied with 100% possible intensity; fog, however, was set to 75%, as this weather condition has such a huge impact on visibility for algorithms relying primarily on data gathered from video frames.

In this analysis, tracking methods were evaluated based on parameters present in MOTChallenge given in Table 3.

The most complex parameter for comparison is MOTA (multiple-object tracking accuracy), which was introduced in Table 3 to describe algorithm accuracy. The MOTA parameter could be less than zero when the sum of errors exceeds the total number of objects able to be tracked in a scene. Although this parameter describes the overall performance of an algorithm, it by itself is not a sufficient measure to describe an algorithm’s performance. Therefore, MOTP (multiple-object tracking precision) was introduced to calculate the overall precision of an algorithm. This parameter typically showed a small variation across different algorithms but was usually between 69.6% up to 71.6%.

### 2.3. Tracking Algorithms

The reliability of the approach described herein was tested by application of several tracking algorithms. The primary requirements of the proposed method are the availability of results in MOTChallenge and of the algorithms’ source code. The tracking algorithms evaluated were as follows:

- High-speed tracking by detection based on intersection over union (IOU) by Erik Bochinski et al. [28] and its further extension [29] in which authors based their tracking algorithm overlap. Method was tested on DETRAC dataset [30].
- Tracking by detection (TBD) by Andreas Geiger [6], in which multiple-object tracking is performed in three stages. All detections are correlated with detections from consecutive frames using bounding box overlap and appearance. A Kalman filter is used in predictions, and detections are than matched between frames using the Hungarian method for bipartite matching. In order to reduce the number of missed detections caused by gaps or occlusions, the author of TBD also employed associated tracklets. TBD was originally tested on author’s own dataset.
- Tracklet Confidence (TCF) by Seung-Hwan Bae and Kuk-Jin Yoon [31], who based the method on tracklet confidence so as to handle track fragments during unreliable detections and occurrence of occlusions and to attain online discriminative appearance learning to avoid errors. For the performance evaluation, the authors used the following datasets: CAVIAR [32], PETS09 [1], and ETH Mobile scene [33].
- Enhancing linear programming (ELP) with Motion Modeling for Multi-target Tracking [34]. During the detection phase, this algorithm employs the authors’ pedestrian detector, creating a group of detections in the form of bounding boxes. After that, all previously detected objects are gathered to create tracklets which include every object and form a network. Method was originally evaluated on Oxford town center [35] and PETS09 [1].
- High density homogeneous (HDH) [36]. Unlike most object detectors, this one requires no object texture or image learning. The detection algorithm localizes targets based on local maxima search, and tracking is based on a greedy, graph-based method, which matches objects with short tracks and performs backward validation in time windows. Method was verified on ETH [33], PETS09 [1] and TUD [37].
- Discrete continuous energy (DCT) [38]. This approach incorporates both data associations and trajectory estimations in one objective function. The biggest benefit from this approach is that the continuous factor allows many trajectory properties to be modeled that a regular discrete formulation would not be capable of capturing. This method gathers all unlabeled detections and then creates possible trajectory hypotheses to re-estimate those trajectories using discrete-continuous optimization. Method was evaluated on ETH [33], PETS09 [1] and TUD [37].

## 3. Results

### 3.1. Crowd Density

**Figure 5:**
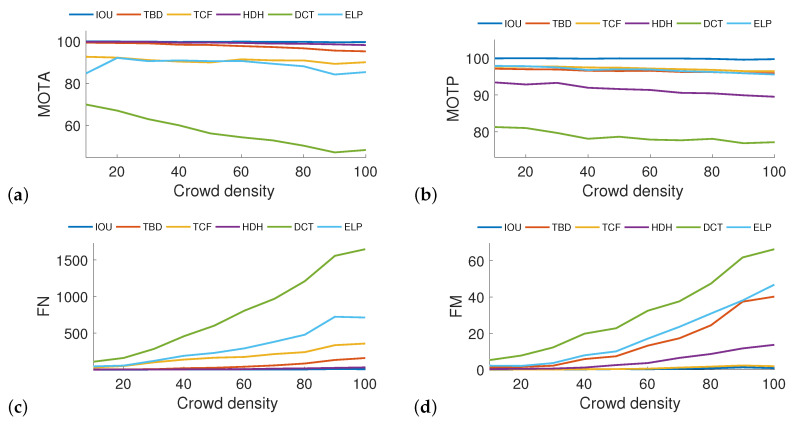
The influence of crowd density on four parameters (MOTA (**a**), MOTP (**b**), FN (**c**), and FM (**d**)) with respect to the total number of pedestrians in the simulations (from 10 to 100 in increments of 10). As can be seen above, the values of FN and FM increase as the number of pedestrians increases, whereas MOTA and MOTP changes slightly. More efficient methods can easily be found. The IOU method provided the most stable and accurate results, whereas the DCT method yielded the worst results, which were not adjusted to simulated data.

**Figure A2:**
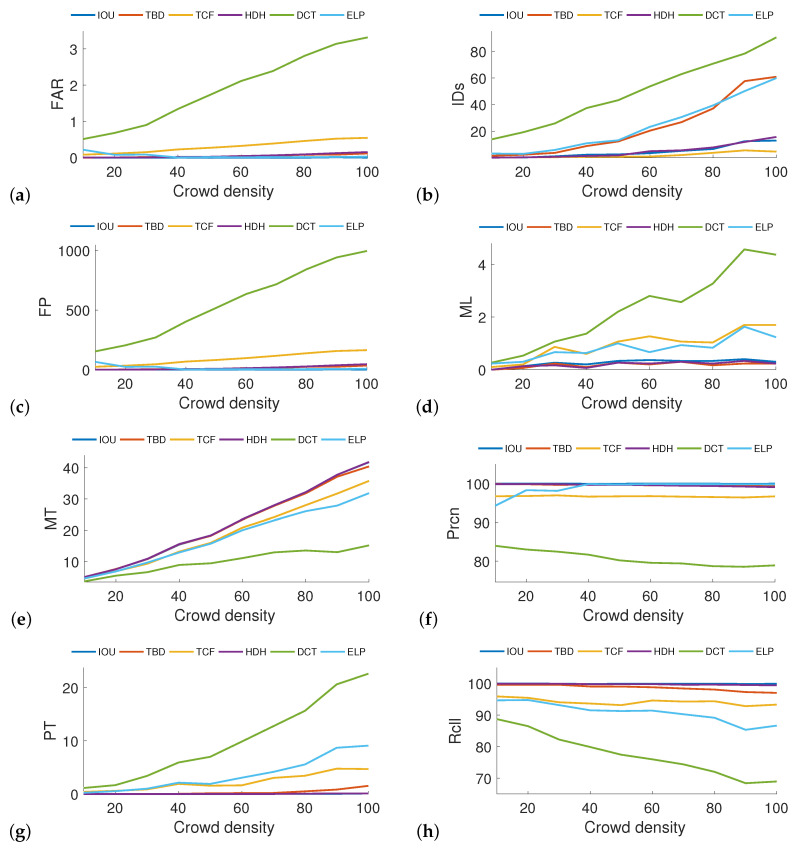
The influence of crowd density on remaining parameters with respect to the total number of pedestrians on simulations (from 10 to 100 with step 10) for FAR (**a**), IDs (**b**), FP (**c**), ML (**d**), MT (**e**), Prcn (**f**), PT (**g**), Rcll (**h**).

The first part of the tests was performed sought to verify the impact of crowd density. The tracking algorithms were evaluated based on changes in crowd density, which began with 10 pedestrians and then increased to 100 pedestrians in increments of 10. Lighting conditions were not affected by additional post-processing methods. Results of the testing are shown in Figure 5 for four different parameters—MOTA, MOTP, FN, and FM. (All remaining parameters can be found in Appendix A Figure A2). The values of almost all parameters (FM, IDs, FAR, FN, FP, ML, MT, PT) exhibited near-linear increases as the number of pedestrians increased, as was expected. In this situation, the most efficient methods have the lowest parameter values. In contrast, the parameters Prcn, Rcll, MOTA, and MOTP take on higher values for relatively more efficient methods. These parameters are influenced by the number of objects ground truth, and the relations are more constant. On the other hand, a good comparison can be observed between parameters MT and ML, for which for mostly tracked the higher value is desired (IOU had the highest overall score), whereas in mostly lost the lower value is expected (IOU had the lowest overall value). Among the tested tracking methods, the IOU algorithm garnered the best performance, and the DCT algorithm the worst. The reason for the latter was that the search for optimal values of the DCT’s internal parameters was out of scope. The other algorithms did not require additional optimization.

**Figure 6:**
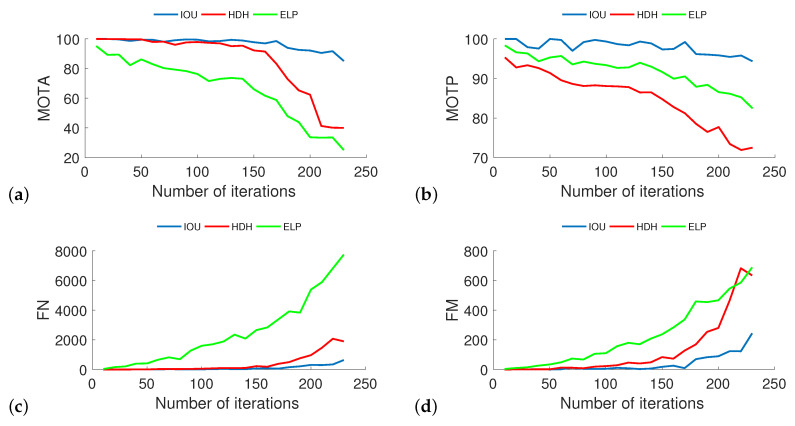
The influence of deeper analysis of crowd density on four parameters (MOTA (**a**), MOTP (**b**), FN (**c**), and FM (**d**)) with respect to the total number of pedestrians in the simulations (from 10 to 230 in increments of 10). As can be seen above, the values of FN and FM increase as the number of pedestrians increases, whereas MOTA and MOTP decrease for number of pedestrians higher than 100.

Due to the extended computation time of some algorithms, full analysis for a larger crowd was only possible for three methods reported in Figure 6. Other methods did not scale well with increasing crowd density. They required even several hours to analyze a single video stream, so they were omitted. Performed calculations show that with the increasing crowd numbers in the simulation, the effectiveness of all compared methods decreases, but the order of their effectiveness does not change compared to previous analyzes. As seen in Figure 6, the values of the MOTA parameter fall to 85% (IOU), 40% (HDH), 25% (ELP) and MOTP parameter to 94% (IOU), 82% (ELP) and 72% (HDH).

### 3.2. Weather Conditions

**Figure 7:**
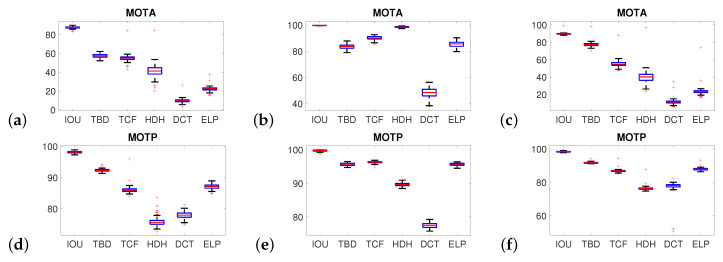
The influence of weather conditions on the MOTA (first row) and MOTP (second row) parameters under rain (**a**,**d**), snow (**b**,**e**), and fog/steam (**c**,**f**). The main differences can be observed in rain and fog conditions while the smallest impact on results has snow conditions.

**Figure 8:**
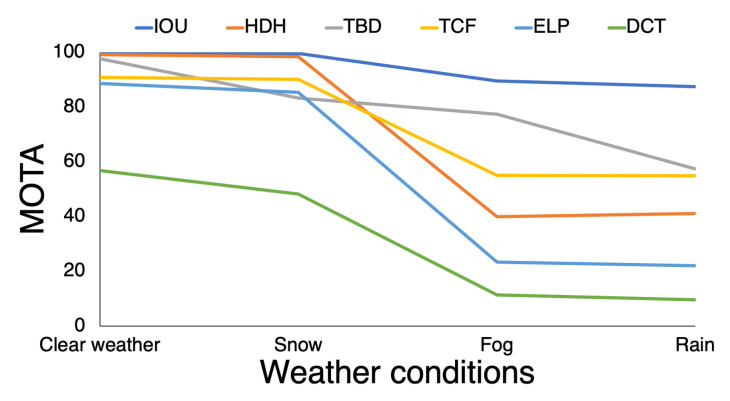
MOTA parameters for all methods with respect to (1) clear weather, (2) snow, (3) fog and (4) rain. It can be observed that some methods (ELP, HDH and TCF) generate better results in clear weather but being influenced by additional weather effects they failed.

**Figure A3:**
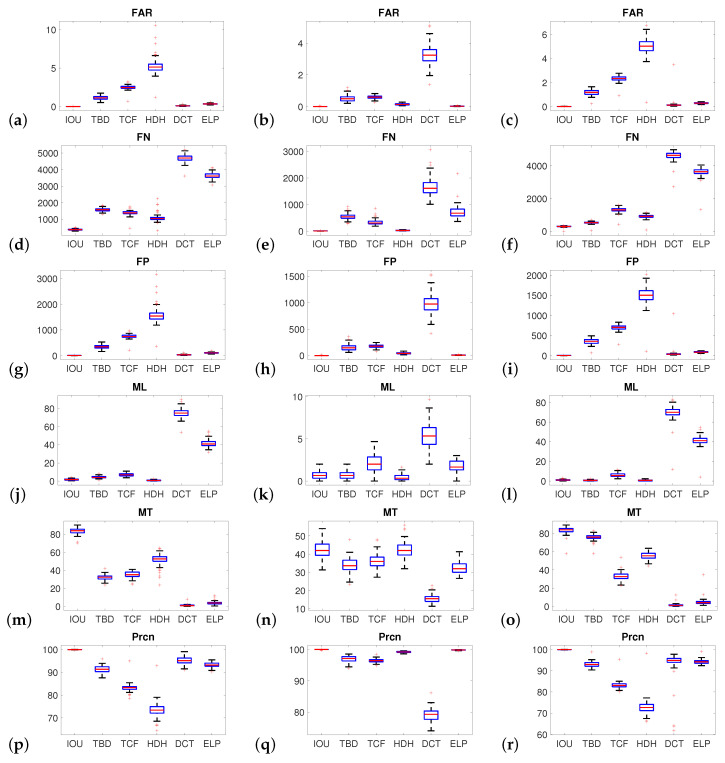
The influence of weather conditions on remaining parameters for rain (first column), snow (second column) and fog/steam (third column) in the form of boxplots for FAR (**a**–**c**), FN (**d**–**f**), FP (**g**–**i**), ML (**j**–**l**), MT (**m**–**o**), Prcn (**p**–**r**).

The primary benefit of performing crowd simulations is the potential to validate different weather conditions, the influence of lighting conditions, and other artifacts. In validating the algorithms, three weather conditions were employed: (a) rain with clouds (first column), (b) snow with clouds (second column), and (c) fog (steam) with 75% intensity (third column). Each tracking algorithm was tested a sufficient number of times (one hundred times), thus ensuring repeatability of methods. Figure 7 presents the MOTA and MOTP parameter results, in the form of boxplots, for each of the three weather conditions. (The remaining parameters are given in Appendix A Figure A3). As can be seen, the rain and fog/steam conditions show the primary differences. The results for snowy conditions (second column) show smaller variability and decreased influence on efficiency. The most efficient method is IOU and the least efficient is the DCT for the same reason as with crowd density. Additionally, in Figure 8 the influence of different weather conditions: (1) clear weather, (2) snow, (3) fog and (4) rain on MOTA parameter for all methods is presented. It is visible that the most challenging weather conditions are fog and rain. In contrast snow has low influence on the results and clear weather gives the highest MOTA value.

### 3.3. Comparison to MOTChallenge

The last step evaluating the algorithms was to compare the results of MOTChallenge [3], which is a benchmark that introduced a true revolution in the multi-object tracking benchmark field. The authors of MOTChallenge introduced video material datasets of a size never before equaled. Its dataset encompasses a wide variety of lightning conditions, crowd densities, and viewpoints. The dataset was augmented by data from previously introduced datasets like PETS. The idea of the benchmark is not to test how good the algorithm performs on a given video sequence but how it performs on a diverse set of sequences. For each sequence available in the MOTChallenge dataset, the benchmark provides a detection file and a ground truth file that were created by the community in order to avoid mistakes that would cause the benchmark to be unreliable. Everyone wishing to test an algorithm against the “challenge” prepared by the authors of the proposed system is free to download the training and benchmark data and submit the results thereafter. These results will then be placed in the ranking, which is reset every year, usually with some additional sequences added to the benchmark. Every participant can also submit his algorithm’s code together with a link to any related publications. In recent years, the MOTChallenge authors have also released the modified version of the benchmark, which allows the testing of algorithms capable of object detection only. Researchers standing behind the MOTChallenge are continuously releasing newer version of the benchmark. Since its inception, they have released the following: 2D MOT 2015, 3D MOT 2015, MOT16, MOT17Det, MOT17, CVPR 2019 Tracking Challenge, and CVPR 2019 Detection Challenge. As every new version contains datasets from previous releases and contributes new ones, the dataset is constantly growing.

Data on the MOT ranking from different years were collected and evaluated methods were verified. Specifically, results from MOTChallenge were compared to mean values of different parameters of crowd simulations from the crowd density, rain, snow, and fog/steam tests. The main purpose of this comparison was to compare the different algorithms’ results from the simulator with the actual MOT ranking. In order to compare a few approaches to data validation, the MOTA (Table 4) and MOTP (Table 5) parameters were calculated. In general, method IOU provided the best results for the MOTA parameter with method TBD yielding the second-best results. (HDH was not classified in MOTChallenge and, under snow conditions, TCF, ELP, and TBD exhibited slight differences.) In MOTChallenge on the first place of ranking DCT method can be found; however, for crowd simulations, with no modification of the parameter source code, that method fails and so was placed in last position. The main differences and variations in method results can be observed in rain and fog/steam conditions. Those weather conditions gave the different methods the biggest difficulties; only small deviations were apparent in snowy conditions (i.e., the final images were not disturbed by snow in a satisfying way) and a potential user could test the behavior of an algorithm with respect to crowd density by changing number of pedestrians.

## 4. Discussion

The experience coming from using automated, crowd simulation-based approach to video tracking evaluation is its flexibility and ease of implementation. Evaluation scenarios can be very easily planned, data are readily presented to video tracking algorithms leading to reliable scorings and ordering of algorithms. CrowdSim tool allows comprehensive evaluation and prioritization of any available state-of-the-art method of video tracking. Here 6 methods have been evaluated which were developed over 2013–2017. That choice was dictated by availability and functionality of the tested tools. The authors of some of the methods available in the latest MOT have not released the source code [39,40,41,42,43,44,45,46,47,48]. Some of the available methods were compiled but unfortunately required additional code modifications, input format adjustments, or generated erroneous results [49,50,51,52].

Important aspect of accessing presented method was comparison to existing approaches, mainly to presently most commonly applied MOTChallenge database. For easy situations obtained ordering of video tracking algorithms highly coincides with MOT ranking. However, scenarios which are presently poorly covered by manually prepared data, rainy, foggy or snowy condition can strongly influence evaluations. IOU method, which achieves top MOT scores, for data generated by CrowdSim is also most efficient. Increasing number of pedestrians or adding rain or snow effect decreases scoring of all compared methods below 90. CrowdSim tool for benchmarking tracking algorithms generates video streams, which are less realistic than real images e.g., obtained from monitoring systems. However, in contrast to real-world, simulated images are much more repeatable and have all parameters under control. Therefore, despite the gap in their reality, they allow drawing more robust conclusions concerning quality of tracking algorithms, as it has been shown in experiments. Clearly, by advancing proposed technology of generating artificial images, in the future versions the authors can come closer to the reality of simulated scenes.

The performed analyses imply that the MOTChallenge ranking cannot be treated as a reference data for all situations. Using collections of video streams prepared for this ranking does not allow for controlling important parameters affecting efficiency of tracking, light conditions number of pedestrians, weather conditions. The data available in the MOTChallenge are too sparse to study differences in values of tracking efficiency as functions of these parameters. The MOTChallenge scorings yielded lower values of the MOTA and MOTP parameters in terms of crowd simulations. For MOTP, the variability in results is much lower than for the MOTA parameter.

### 4.1. Application of the Concept of Crowd Simulations

Proposed system of automatic generation of different scenes ensures that tracking methods designers cannot overfit their parameters by targeting only limited sets of real videos. Examples of a crowd simulator show that it can be applied to verify and validate tracking algorithms. The main benefit of such an approach is in the wider possibility of comparison and generation of different situations for a scene. The CrowdSim enables verification of the impact of crowd density (i.e., the number of pedestrians in a scene) on tracking algorithms. In general, it can be observed that tracking methods suffered from a greater number of problems with increased numbers of pedestrians. On the other hand, the influence of three different weather conditions (as well as associated light) on the quality of tracking was checked and it was observed that the main difference occurred within the rain and fog conditions. Obtained results demonstrated the main trends with respect to parameters, and the idea of crowd simulations in tracking evaluation can be developed and performed under conditions of increasing sophistication. Although given approach was compared with the results from MOTChallenge, the main goal was not to obtain exactly the same parameter values, an impossibility. MOTChallenge contains videos created under different conditions, which were presented in the rankings in averaged form. On the other hand, not all methods were given in each ranking, and there were only a few working open-source codes (even for those methods which topped rankings). In method verification, the authors focused primarily on the MOTA and MOTP parameters, which are the most complex. It was possible to check the methods that yielded repeatable results and the conditions in which algorithms gave different results, and finally the efficiency of the tracking methods included in the analysis was verified.

To reach a compromise between the quality of simulations and the computation time of tracking methods, it was decided to choose 10 FPS and the resolution of 800 × 600. The main intention was to verify the influence of crowd density and weather conditions, so the number of repetitions is much higher than in single videos in MOT. Current video surveillance systems range from 10–25 FPS of quality but still many popular benchmarks have similar parameters (Kitti [53]—10 FPS, PETS09 [1]—7 FPS). Lower FPS value is still dictated by video storage and data transmission. In IPVM report [54] it can be observed that average frame rate in video surveillance increased into 15 FPS in 2019 (40% of 11–15 FPS and 28% of 6–10 FPS) from 10 FPS in 2016 (51% of 6–10 FPS and 32% of 11–15 FPS). Furthermore, the group from University College London proved that the minimum frame rate level to detect events in similar video systems is 8 FPS [55]. The authors of [56] treat the real-time efficiency in the value of 10 FPS. Nevertheless, CrowdSim enables modification and increase (or decrease) of either frame rate or image resolution so that potential user can adjust images to their needs.

### 4.2. Future Works

The proposed solution solves many complex problems but nonetheless requires additional development in the future. For the creation of human movement, built-in tools were used that are available in Unity and external tools. Nevertheless, the simulation must be equipped with some additional mechanism capable of making human movement more natural with limited possibilities of occlusions. In the case of action recognition and more advanced simulation of the movement of individual agents, it is planned to use an additional animation library, as well as record own animations in motion capture systems. It should be considered replacing the navigation mesh with planned movement in which all behavior is planned a priori and is simulated by the software. An additional element that makes the simulations real is the addition of a background in the form of other moving objects that do not participate in the tracking process. There are also plans to prepare first-person images that could move with the crowd. A scene should consist of more realistic elements including traffic jams in the example of moving cars. The crowd simulations themselves should be more complex, consist of more differently looking people, and generate more problems for tracking algorithms. In order to improve the reality of scenarios such as weather conditions future versions of simulator should be equipped with different kind of post-processing methods as well as shaders implemented for that case.

## 5. Conclusions

In this paper, a novel approach to validation of video tracking algorithms was proposed. The crowd simulation system for video tracking benchmarking was prepared in a game engine environment. Previous approaches used real-world collections of video streams, catalogued and annotated manually. The proposed methodology was inspired by problems, which follow from weaknesses of manual preparation schemes mentioned in several literature references and commonly encountered in preparation and validation of real-world surveillance systems, limited volume and resolution of data streams, insufficient replicability, problems in planning and implementing desired scenarios.

In the proposed algorithmic solution and elaborated implementation “CrowdSim”, resulting images are automatically annotated in the commonly used convention and can be readily applied to evaluation of tracking algorithms. The output of “CrowdSim” reproduces formats and standards, in particular those of MOTChallenge, already in use for benchmarking video surveillance systems. All output indexes for quality of tracking are reliably reproduced. Simulation scenarios can represent variety of situations and can be controlled by parameters such as time of day, crowd level, weather conditions, and background. Parameters can appear with different levels. Studies on sensitivities of algorithms to different variants, different levels of values of disturbing parameters can easily be conducted.

To confirm the usefulness of the proposed approach several tests were conducted concerning comparisons of applications of different tracking algorithms (IOU, TCF, ELP, TBD, HDH, DCT) to the generated data. Significant improvements were demonstrated by comparison of the described approach to existing benchmarking systems based on manual annotations. CrowdSim made possible exhaustive comparisons of the studied tracking algorithms with respect to several important indexes, crowd density, weather conditions (rain, snow, fog/steam), lighting conditions, time of day. Significant differences in responses to changes in the indexes between video tracking systems were observed, ranging one order of magnitude for MOTA parameter. In contrast, manually annotated systems are likely to flatten ranges of quality indexes of compared algorithms due to insufficient variability of parameters.

Several directions of future development of the elaborated system are possible, improving realism of generated images, increasing variability ranges of parameters, combining different indexes in video streams. Developing new versions of crowd simulators will contribute to improvements in efficiency of the constructed surveillance systems. CrowdSim is freely available at the dedicated webpage (https://crowdsim.aei.polsl.pl) along with several simulated benchmark data sets.

## Figures and Tables

**Table 1:** Detailed information on the crowd-density dataset.

Parameter	Details
Number of pedestrians	10–100 (step 10)
Frame rate	10 FPS
Length	30 s
Number of cameras	3
Total number of sessions	300

**Table 2:** Detailed information on the weather dataset.

Parameter	Details
Number of sessions per weather condition	100
Number of pedestrians	100
Number of weather conditions	3
Frame rate	10 FPS
Length	30 s
Number of cameras	3
Total number of sessions	900

**Table 3:** Detailed information on MOTChallenge parameters employed in comparison.

Parameter	Details
PT (partially tracked)	The number of objects tracked for more than 20% but less than 80% of their presence in a scene
MT (mostly tracked)	The number of objects tracked for more than 80% of their presence in a scene
ML (mostly loosed)	The number of objects tracked for less than 20% of their presence in a scene
FM (fragments)	The number of trajectories with one or more gaps in their tracklets.
IDs (ID switches)	One object occluding another so that the tracking algorithm continues tracking the wrong object
FP (false positive)	An instance where an algorithm identified a trajectory but could not relate it to an existing object
FN (false negative)	Describes the number of objects not tracked by an algorithm
GT (ground truth)	Number of objects described in the reference file
Recall	Ratio between objects that were possible to track (GT) and those actually tracked
Precision	Relationship of correct trajectories to the total number of possible trajectories
FAR	Number of false positives relative to the total number of frames comprising a tested sequence
MOTA	Three sources of error: false negatives, false positives, and ID switches.
MOTP	Average difference between true positives and corresponding ground truth targets.

**Table 4:** Comparison of crowd simulation results under different conditions and MOTChallenge ranking with respect to MOTA parameters. The IOU and TBD methods produced the best results. The biggest differences are visible for rain and fog conditions and within each of these conditions, the biggest differences are apparent.

Crowd Density		MOTChallenge		Rain		Snow		Fog/Steam	
Method	Average	Method	Average	Method	Average	Method	Average	Method	Average
IOU	99.8	IOU	57.1	IOU	87.5	IOU	99.6	IOU	89.6
HDH	99.2	TBD	33.7	TBD	57.5	HDH	98.5	TBD	77.4
TBD	97.7	DCT	33.2	TCF	54.9	TCF	90.1	TCF	55.1
TCF	90.9	ELP	25	HDH	41.1	ELP	85.4	HDH	40
ELP	88.7	TCF	15.1	ELP	22.1	TBD	83.4	ELP	23.4
DCT	56.9	HDH	n/c	DCT	9.6	DCT	48.3	DCT	11.4

**Table 5:** Comparison of crowd simulation results under different conditions and the MOTChallenge ranking with respect to MOTP parameters. The IOU method again produces the best results; however, in comparison to the MOTA parameter, the differences in results are smaller.

Crowd Density		MOTChallenge		Rain		Snow		Fog/Steam	
Method	Average	Method	Average	Method	Average	Method	Average	Method	Average
IOU	99.9	IOU	77.1	IOU	98.2	IOU	99.7	IOU	98.3
TCF	97.2	TBD	76.5	TBD	92.4	TCF	96.3	TBD	91.7
ELP	96.8	DCT	75.8	ELP	87.1	TBD	95.7	ELP	87.9
TBD	96.5	ELP	71.2	TCF	86.1	ELP	95.7	TCF	86.8
HDH	91.5	TCF	70.5	DCT	77.9	HDH	89.6	DCT	77.2
DCT	78.6	HDH	n/c	HDH	76	DCT	77.5	HDH	76.3

## Abbreviations

The following abbreviations are used in this manuscript:

PT	Partially Tracked
MT	Mostly Tracked
ML	Mostly Loosed
FM	Fragments
IDs	ID switches
FP	False Positive
FN	False Negative
GT	Ground Truth
Rcll	Recall
Prcn	Precision
FAR	False positives Relative to the total number of frames
MOTA	Multiple-Object Tracking Accuracy
MOTP	Multiple-Object Tracking Precision
IOU	High-speed tracking by detection based on Intersection Over Union
TBD	Tracking By Detection
TCF	Tracklet Confidence
ELP	Enhancing Linear Programming
HDH	High Density Homogeneous
DCT	Discrete Continuous Energy
FPS	Frame Per Second

## Appendix A

### Appendix A.1. CrowdSim—Examples

The different examples of weather conditions influencing the results of crowd simulations are visible in Figure A1.

### Appendix A.2. Crowd Density

The remaining parameters showing the influence of crowd density on accuracy of evaluated algorithms are presented in Figure A2.

### Appendix A.3. Weather Conditions

The remaining parameters showing the influence of weather conditions on accuracy of evaluated algorithms are presented in Figure A3.
